# Inflammatory markers and noncoding‐RNAs responses to low and high compressions of HIIT with or without berberine supplementation in middle‐aged men with prediabetes

**DOI:** 10.14814/phy2.16146

**Published:** 2024-08-06

**Authors:** Mehdi Nikseresht, Valiollah Dabidi Roshan, Khadijeh Nasiri

**Affiliations:** ^1^ Department of Exercise Physiology, Faculty of Sport Science University of Mazandaran Babolsar Iran; ^2^ Athletic Performance and Health Research Centre, Faculty of Sport Science University of Mazandaran Babolsar Iran

**Keywords:** Berberine, H19, HIIT, insulin resistance, interleukin‐1β, NLRP3

## Abstract

This study compared the capacity of two different models of HIIT [high‐(HC) and low‐(LC) compression], with or without the use of berberine (BBR), on NOD‐like receptor pyrin domain‐containing protein‐3 (NLRP3), H19, interleukin (IL)‐1β, high‐sensitivity C‐reactive protein (hs‐CRP), and insulin resistance markers. Fifty‐four middle‐aged men with overweight or obesity and prediabetes [fasting blood glucose (FBG) 110–180 mg/dL] were randomly and equally assigned to the HC, LC, HC + BBR, LC + BBR, BBR, and non‐exercising control (CON) groups. The HC (2:1 work‐to‐rest) and LC (1:1 work‐to‐rest) home‐based training programs included 2–4 sets of 8 exercises at 80%–95% HRmax, twice a week for 8 weeks. Participants in the berberine groups received approximately 1000 mg daily. All exercise interventions led to a significant reduction in hs‐CRP, IL‐1β, insulin, FBG, and insulin resistance index (HOMA‐IR) versus CON. Notably, there was a significant reduction in FBG and HOMA‐IR with the BBR group compared to the baseline. Both NLRP3 and H19 experienced a significant drop only with LC in comparison to the baseline. While both exercise protocols were beneficial overall, LC uniquely exhibited more anti‐inflammatory effects, as indicated by reductions in H19 and NLRP3. However, the addition of berberine to the exercise programs did not demonstrate additional benefits.

## Graphical abstract

Two different models of HIIT effects on inflammatory markers.

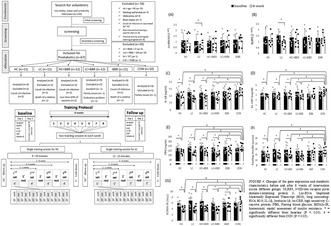

## 1 INTRODUCTION

Obesity is an important risk factor for many chronic diseases, including metabolic syndrome and type 2 diabetes (T2D). This is because circulating levels of pro‐inflammatory cytokines, such as interleukin (IL)‐1β and C‐reactive protein (CRP), are increased in individuals with obesity (Liu & Wang, 2021; Nikseresht et al., 2016). It has been recognized that NOD‐like receptor pyrin domain‐containing protein 3 (NLRP3) is a trigger point for the activation of these mediators (Wani et al., 2021). Additionally, long noncoding RNAs (LncRNAs), which are noncoding genes with more than 200 nucleotides, play an important role in regulating NLRP3 inflammasome activity (Menon & Hua, 2020). For example, H19, one of the LncRNAs, is involved in the regulation of cardiovascular function, and its expression is associated with metabolic syndrome and atherosclerosis (Farsangi et al., 2021; Shi et al., 2020). However, H19 can decrease the secretion of IL‐1β and IL‐18 in mouse muscle tissue by inhibiting NLRP3, leading to improved insulin sensitivity (Han et al., 2021). It has been reported that LncRNA H19 may positively affect metabolic disorders, especially in the early stages (Qin et al., 2019). Notably, the level of H19 is lower in obese individuals with high glucose (Ghafouri‐Fard & Taheri, 2021). This reduction may be induced by high‐fat diets, which have been shown to reduce H19 in mice (Duan et al., 2018; Geng et al., 2018). Another study found that H19 expression significantly decreased after myocardial infarction in rats but returned to normal levels after a 4‐week endurance training period (Farsangi et al., 2021). Additionally, H19 plays a critical role in maintaining all types of slow muscle fibers and exercise endurance (Yue et al., 2023). However, the response of this marker to exercise in humans has not yet been investigated.

In recent years, the use of high‐intensity interval training (HIIT) has expanded to maintain and improve the health of people of different ages. Many researchers have confirmed the beneficial effects of HIIT on various organs of the body (Chin et al., 2020; Keech et al., 2020). While this is not always the case, it is believed that HIIT might be a good choice because it can be performed in less time and is often more effective than moderate‐intensity continuous training (MICT), particularly in reducing insulin resistance markers and hemoglobin glycated (HbA1c) in patients with T2D and metabolic syndrome (Viana et al., 2019). Additionally, HIIT is a time‐efficient alternative to MICT for improving metabolic health in older individuals, as it has shown more favorable regulation of anti‐inflammatory activity compared to MICT (Sun et al., 2020). Among its different models, low‐volume HIIT has been shown to improve some pro‐inflammatory and metabolic biomarkers (Chin et al., 2020; Leggate et al., 2012). For example, a very recent study indicated that 12 weeks of low‐volume HIIT had a greater positive effect on IL‐6 and CRP compared to whole‐body electromyostimulation and resistance training in people with metabolic syndrome (Reljic et al., 2022). However, another study demonstrated that both MICT and HIIT had beneficial effects on IL‐1β and NLRP3 expression in young men (Khakroo Abkenar et al., 2020).

As a naturally occurring benzylisoquinoline alkaloid, berberine (BBR) has a long history of medical applications in traditional Chinese medicines. It is primarily found in the roots, rhizomes, and stem bark of various medicinal plants from the Ranunculaceae, Rutaceae, and Berberidaceae families. It has been confirmed to exhibit various clinically useful biological properties, including anti‐cardiovascular disease and anticancer effects (Ai et al., 2021). As a traditional herbal medicine with anti‐inflammatory effects and the ability to improve insulin resistance, BBR has attracted significant attention for treating T2D (Guo et al., 2021). Additionally, compared to the popular diabetic drug metformin, BBR is a potent oral hypoglycemic agent with beneficial effects on lipid metabolism (Yin, Xing, & Ye, 2008). For instance, Paul et al. (2019) reported that consuming BBR had a protective effect against platelet aggregation, apoptosis, and superoxide production by attenuating oxidative stress (Paul et al., 2019). BBR can specifically block NEK7‐NLRP3 interaction by targeting NEK7 and sequentially inhibit IL‐1β release, independent of NF‐kB and TLR4 signaling pathways (Zeng et al., 2020). To date, it has demonstrated antiarrhythmic, antiapoptotic, and anti‐inflammatory effects as well as a reduction in the size of infarcted myocardium (An et al., 2022; Banaei et al., 2020). Further research is necessary to investigate the effects of BBR combined with exercise training on inflammatory markers due to the lack of human‐based evidence. It is hypothesized that combining low‐volume HIIT with BBR may be a better scheme to improve inflammation. Additionally, the inflammatory responses to different models of HIIT may vary.

Thus, the main purpose of this study was to investigate the effects of 8 weeks of low‐volume HIIT with low compression (LC) versus low‐volume HIIT with high compression (HC), with or without BBR, on the regulation of noncoding RNAs H19, NLRP3, IL‐1β, hs‐CRP, and insulin resistance markers in middle‐aged men with overweight or obesity and prediabetes.

## 2 MATERIALS AND METHODS

### 2.1 Experimental approach to the problem

We designed a single‐blinded, randomized controlled study to compare the effects of 8 weeks of LC versus HC with or without daily BBR supplementation on the expression of LncRNA and coding RNA inflammatory genes expression, IL‐1β, hs‐CRP, insulin resistance markers, and functional capabilities in middle‐aged men with overweight or obesity and prediabetes. Participants were first matched by aerobic fitness, age, and BMI, and then randomly assigned into the following groups: HC, LC, HC + BBR, LC + BBR, BBR, and non‐exercising control (CON) groups.

Participants in the training groups performed 8 weeks of HIIT with different compression levels, while participants in the CON and BBR groups maintained a sedentary lifestyle throughout the period. The two exercise models were identical, except that the rest period between sequences in the HC protocol was half that of the other model. Participants were familiarized with all procedures before any tests were conducted. All tests were performed at the same time of day by the same researcher to account for circadian rhythms.

Physiological, functional, and anthropometric assessments were measured before and after the 8‐week intervention. Blood samples were taken 3 days before the first session and 3 days after the last session to determine serum levels of IL‐1β, hs‐CRP, insulin, glucose, and the inflammatory markers NLRP3 and H19.

### 2.2 Participants

**FIGURE 1:**
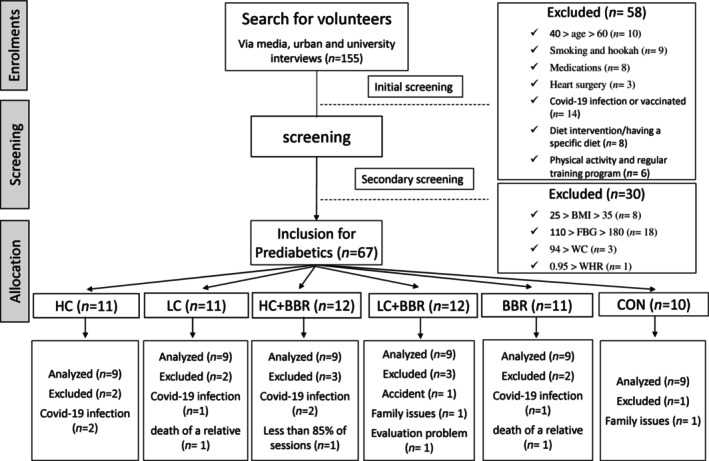
Flowchart of this study.

Sixty‐four middle‐aged men with overweight or obesity and varying glycemic levels participated in this study. None of the participants had contracted COVID‐19 or received any COVID‐19 vaccine. Additionally, they did not have a history of chronic diseases, smoking, regular medication use, or adherence to a specific diet (only those following an omnivorous diet were included). They continued a sedentary lifestyle, engaging in less than 60 min of physical activity per week during the past 6 months. Participants who did not attend at least 13 out of 16 training sessions were excluded from the training groups (Figure 1). All participants signed an institutionally approved informed consent document before the start of the study. This study was approved by the Research Ethics Committee at the University of Mazandaran (IR.UMZ.REC.1401.011).

### 2.3 Anthropometry

After fasting for 10 h, participants' body weight and height were measured with minimal clothing using a balance scale (Seca700 Mechanical Column Scale, Birmingham, UK) calibrated to the nearest 0.1 kg and 0.1 cm. Waist circumference and waist‐to‐hip ratio were measured using a measuring tape with an accuracy of 1 mm. BMI was then calculated [body mass (kg)/height (m^2^)]. Lean body mass and body fat percentage were assessed by an expert using a standard technique (Medigate Inc., Boca X1, South Korea). Before the test, they were asked to stand in the designated position on the device with minimal clothing and without metal and electrical equipment.

### 2.4 Functional capability

#### 2.4.1 Maximum oxygen consumption (VO_2_ max)

The exercise time to exhaustion was measured using Broce's exercise protocol and then used to assess VO_2_ max with the following formula: VO_2_ max (ml/kg/min) = 14.76 − (1.379 × T) + (0.451 × T^2^) − (0.012 × T^3^). After a 10‐min warm‐up, all participants performed the test on a treadmill under the supervision of an expert. During the test, heart rate (HR) was monitored using a standard device (Polar®, Finland). The test was stopped when each subject reached 90% of their heart rate maximum (HRmax = 220 − age) or displayed some symptoms such as ataxia and dizziness. All tests were performed at a temperature of 24 to 26°C and a relative humidity of 50% to 60%.

#### 2.4.2 Hand grip & leg isometric strength

To measure isometric hand and forearm strength, participants pressed the dynamometer grip (DHD‐3, SH1003, SAEHAN, Korea) with maximum effort while standing. Maximal isometric leg strength was also measured using a standard method with an accuracy of 0.1 kg (Danesh Salar Company, Iran).

#### 2.4.3 Myocardial oxygen consumption (MVO_2_)

Calculating MVO_2_ directly is difficult; however, double product [DP = HR × systolic blood pressure (SBP)] can be a good indicator of it (Guensch et al., 2019). Systolic and diastolic blood pressure were measured with a digital sphygmomanometer (Roosmax, Model ME701, and Switzerland) after a 10‐min rest. Mean arterial pressure (MAP) was then calculated using the following formula: [SBP + (2 × DBP)]/3.

### 2.5 Estimated dietary energy intakes

In this study, we included only participants who followed a normal diet consisting of 50%–60% carbohydrate, 20%–30% fat, and 10%–15% protein, as measured by the COMP‐EAT software program (National Analysis System, Version 4, London, UK). Participants were asked to adhere to this well‐balanced dietary program throughout the study. To ensure compliance, they were required to record their daily diet for 1 week during both the 4th and 7th weeks of the intervention. Additionally, they were instructed to follow the same diet 1 day before each sampling (before and after training).

### 2.6 Biochemical analyses

Before and after the training, blood samples (8 mL) were obtained in a resting state after a 10‐h overnight fast and 8 h of sleep between 9 a.m. and 10 a.m. Participants were asked to abstain from any exercise for at least 3 days before sampling (both before and after the intervention). Blood samples were taken at least 72 h after the last exercise session in the training groups. The samples were centrifuged at 3000 rpm for 10 min at 4°C to separate the serum, which was then frozen at −80°C to measure the biochemical variables. Serum levels of IL‐1β and insulin were measured in duplicate by the ELISA method according to the manufacturer's guidelines. IL‐1β was detected using a commercial assay kit (Karmania Pars Gene, Iran), while insulin concentration was measured using a traditional kit (Monobind test system, MN, USA). The lowest diagnostic values were less than 2 pg/mL and 0.1 μU/mL, respectively. The inter‐ and intra‐assay coefficients of variation were less than 5%. Glucose levels were measured using the glucose oxidase procedure (Autoanalyzer system of Hitachi 917, Japan), and insulin resistance was estimated by the Homeostasis Model Assessment (HOMA‐IR = IR (μU/mL) × FBG (mmol/L)/22.5). Additionally, whole mixed blood with EDTA was used to extract RNA for NLRP3 and H19 analysis.

### 2.7 Total RNA extraction and quantitative real‐time

#### 2.7.1 PCR (qRT‐PCR)

Total RNA of LncRNA‐H19 and NLRP3‐coding genes was extracted from whole blood using the Denazist Kit (Tehran, Iran) according to the manufacturer's instructions. The concentration and purity of the RNA were measured by electrophoresis on agarose gel and spectrophotometry (NanoDrop Model NDNM96, Iran). To remove genomic DNA contamination, the extracted RNA was treated with a DNase‐RNase‐free enzyme. The RNA samples were then incubated in a thermocycler at 37°C for 30 min in tubes without DNase‐RNase (Sinaclon, Iran). Complementary DNA (cDNA) synthesis was performed using a Yekta Tajhiz kit (Iran). Primer sequences were designed using Premier 5 software and synthesized with a Bioneer machine (South Korea). The sequences of the forward and reverse primers are shown in Table 1. Relative quantification of gene expression was performed by qRT‐PCR using a commercial SYBR Green kit (Ampliqon, Canada) with a Corbett 6000 Rotor machine in three different temperature cycles. β‐actin mRNA was used as the reference gene. Target gene expression was quantified using the $2^{-\Delta \Delta C_{t}}$ formula. Each sample was analyzed in duplicate, and results are expressed as fold changes relative to the control group.

**TABLE 1:** Primer sequences of selected coding and noncoding inflammatory genes.

Genes	Primers	Sequence	Access number	Base pair
B‐Actin	Forward	CGGGAAATCGTGCGTGAC	NM_001101.5	109
	Reverse	GCTCGTAGCTCTTCTCCAGGG		
NLRP3	Forward	GAGCCTCAACAAACGCTACAC	NM_183395.3	151
	Reverse	ATCGGGGTCAAACAGCAACT		
LncRNA‐H19	Forward	GGAATCGGCTCTGGAAGGT	NR_003491.3	174
	Reverse	TCAGCTCTGGGATGATGTGG		

### 2.8 Training programs and berberine supplementation

**FIGURE 2:**
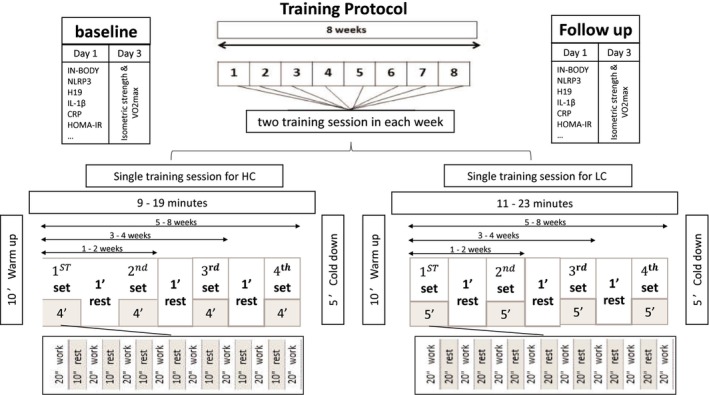
Scheme of the training sessions.

In this study, home‐based training programs were designed due to COVID‐19 limitations, as presented in Table 2 and Figure 2. Firstly, participants were familiarized with the training programs and instructed to avoid other exercises throughout the study. The two exercise programs were identical, performed twice a week for 8 weeks, except for the rest period between sequences. The HC protocol involved 2–4 sets, each consisting of 8 sequences of 20 s of physical activity followed by 10 s of rest, with a 1‐min rest between sets (Tabata, 2019). In contrast, the LC protocol included 20 s of rest between sequences. The training intensity for these programs ranged from 7 to 9 on the modified Borg scale, equivalent to 85%–95% of HRmax (Carvalho & Guimarães, 2010; Mabele et al., 2019). The main researcher conducted training sessions with participants via WhatsApp.

**TABLE 2:** Periodized high‐intensity interval training programs.

Programs	Training variables	Week 1–2	Week 3–4	Week 5–6	Week 7–8
HC‐HIIT	Number of sets	2	3	4	4
	Sets × rest (min)	4 × 1	4 × 1	4 × 1	4 × 1
	Duration (min)	10	15	20	20
	Intensity (RPE‐10 scale)	7	7–8	7–8	8–9
	S&C exercises	S	S	S&C	S&C
LC‐HIIT	Number of sets	2	3	4	4
	Sets × rest (min)	5 × 1	5 × 1	5 × 1	5 × 1
	Duration (min)	12	18	24	24
	Intensity (RPE‐10 scale)	7	7–8	7–8	8–9
	S&C exercises	S	S	S&C	S&C

*Notes*: The exercise regimen consisted of two phases, each spanning 4 weeks. During the initial 4 weeks, participants engaged in a set of eight exercises, including Air Squats, High Knee, Knee Push up, Mountain Climber, Standard Plank, Butt Kickers, Split, and Skaters. In the subsequent 4 weeks, the exercise routine became more advanced, incorporating eight more complex exercises: squat jump, skier jumping jacks, push up, mountain climber, super plank, jumping jack, lunge, and skaters Jump.

Abbreviations: C, complex; HC, high compression; LC, low compression; S, simple.

Research suggests that a daily dose of 1000 mg is appropriate for obese/overweight, prediabetic, and diabetic individuals, with minimal notable side effects (Wang et al., 2020; Xiong et al., 2020). Thus, in this study, participants from the HC + BBR, LC + BBR, and BBR groups consumed 1000 mg of BBR in capsule form (Omid Parsina Damavand Company, Thorn Research, Iran) with their breakfast and dinner meals (500 mg each time). According to the manufacturer's data, this product is solely extracted from the barberry plant (Xiong et al., 2020).

### 2.9 Statistical analysis

In the study, we utilized the PASS software (version 11; NCSS, LLC, Kaysville, Utah, USA) to estimate the sample size. Based on data from a previous study (Mahmoud et al., 2022), a total of 54 participants (9 participants per group) were calculated to detect differences using a two‐way ANOVA model with a significance level of 0.05, 80% power, and an acceptable effect size. Normality was assessed using the Shapiro–Wilk and Q–Q plates test. Differences between groups and time points were determined using a two‐way ANOVA. In case of a significant interaction (time × interventions), a Bonferroni post hoc test was employed to identify the location of the difference. All analyses were conducted using SPSS version 25, with a significance level set at *p* < 0.05. Fold changes in gene expression were calculated using the $2^{-\Delta \Delta C_{t}}$ method.

## 3 RESULTS

The anthropometric, body composition, and functional parameters of the participants at baseline and after the intervention are presented in Table 3. None of the interventions caused significant changes in anthropometric and body composition variables when compared to CON. However, there were reductions in body weight and BMI after all interventions in comparison to baseline (all, *p* < 0.05). Waist circumference significantly decreased with all interventions, except for BBR, while the waist‐to‐hip ratio reduced only after HC + BBR and LC + BBR (both, *p* < 0.05). Additionally, LC + BBR resulted in a significant decrease in fat mass and body fat percentage (both, *p* < 0.05).

**TABLE 3:** Baseline and after intervention within‐ and between‐group comparison of physical and body composition parameters.

Variables	Groups (*n* = 54)						*p*‐Value			ES	SP
	HC (*n* = 9)	LC (*n* = 9)	HC + BBR (*n* = 9)	LC + BBR (*n* = 9)	BBR (*n* = 9)	CON (*n* = 9)	*t*	*i*	*t* × *i*		
	Mean ± SD	Mean ± SD	Mean ± SD	Mean ± SD	Mean ± SD	Mean ± SD					
Age (year)	48.42 ± 4.68	49.75 ± 8.13	48.75 ± 4.23	48.42 ± 4.39	52.71 ± 7.25	49.14 ± 4.29					
Body weight (kg)											
Baseline	89.01 ± 13.05	89.82 ± 13.43	88.87 ± 7.97	92.48 ± 15.77	91.78 ± 14.40	92.75 ± 9.45	**0.001**	0.194	0.124	0.15	0.48
After 8 weeks	87.00 ± 12.13^a^	87.77 ± 12.31^a^	86.50 ± 8.48^a^	89.90 ± 14.91^a^	88.41 ± 14.40^a^	93.68 ± 10.10					
BMI (kg/m^2^)											
Baseline	29.84 ± 3.57	30.66 ± 3.64	29.41 ± 2.29	30.63 ± 2.46	30.34 ± 4.25	30.60 ± 2.14	**0.001**	0.529	0.115	0.14	0.40
After 8 weeks	29.19 ± 3.28^a^	29.65 ± 3.33^a^	28.70 ± 2.36^a^	29.73 ± 2.40^a^	29.50 ± 3.77^a^	30.97 ± 2.19					
WC (cm)											
Baseline	99.85 ± 6.17	99.50 ± 5.60	98.00 ± 4.48	101.07 ± 6.50	101.28 ± 8.52	101.71 ± 5.52	**0.001**	0.190	0.154	0.16	0.45
After 8 weeks	97.14 ± 4.70^a^	97.05 ± 5.35^a^	95.37 ± 4.03^a^	98.28 ± 6.94^a^	100.33 ± 6.55	102.14 ± 6.41					
WHR											
Baseline	0.95 ± 0.03	0.96 ± 0.04	0.95 ± 0.03	0.96 ± 0.04	0.97 ± 0.04	0.96 ± 0.04	**0.039**	0.150	0.492	0.10	0.28
After 8 weeks	0.95 ± 0.02	0.95 ± 0.04	0.93 ± 0.04^a^	0.94 ± 0.05^a^	0.96 ± 0.02	0.96 ± 0.03					
BF (%)											
Baseline	29.58 ± 3.79	31.25 ± 4.77	29.37 ± 3.74	29.94 ± 2.85	28.84 ± 3.57	28.28 ± 4.31	**0.016**	0.430	0.141	0.18	0.54
After 8 weeks	29.09 ± 3.85	30.61 ± 5.17	29.06 ± 4.45	28.37 ± 2.58^a^	28.99 ± 4.25	29.34 ± 4.97					
FM (kg)											
Baseline	26.74 ± 6.98	28.16 ± 8.38	26.27 ± 5.48	28.89 ± 6.79	26.82 ± 7.39	31.01 ± 6.14	**0.028**	0.613	0.580	0.11	0.30
After 8 weeks	25.70 ± 6.75	28.02 ± 7.62	25.38 ± 5.93	26.56 ± 5.71^a^	26.18 ± 7.47	32.35 ± 6.66					
LBM (kg)											
Baseline	57.67 ± 5.50	58.47 ± 5.92	58.05 ± 3.53	60.72 ± 8.69	60.44 ± 6.70	60.17 ± 6.05	0.055	0.675	0.174	0.17	0.50
After 8 weeks	57.94 ± 5.89	57.83 ± 5.60	57.95 ± 3.70	60.74 ± 9.12	58.68 ± 5.81	59.22 ± 6.24					
VO_2_max (ml.kg^−1^/min^−1^)											
Baseline	33.79 ± 7.12	31.51 ± 4.50	30.97 ± 5.08	32.86 ± 5.73	30.17 ± 5.37	30.93 ± 4.96	**0.001**	0.190	**0.014**	0.30	0.84
After 8 weeks	38.41 ± 6.85^a^, ^b^	35.80 ± 3.94^a^, ^b^	36.14 ± 6.98^a^, ^b^	37.22 ± 8.80^a^, ^b^	32.00 ± 5.40	30.72 ± 4.90					
LIS (kg)											
Baseline	110.28 ± 9.34	109.77 ± 22.06	105.60 ± 28.90	106.71 ± 18.66	107.78 ± 19.26	107.57 ± 19.41	**0.001**	0.228	0.826	0.32	0.14
After 8 weeks	131.57 ± 10.09^a^	127.26 ± 21.24^a^	129.32 ± 22.44^a^	128.51 ± 15.95^a^	112.57 ± 13.72	111.52 ± 17.04					
Hand grip strength (kg)											
Baseline	42.57 ± 3.86	40.61 ± 8.05	39.64 ± 6.48	39.38 ± 6.32	38.54 ± 6.83	42.08 ± 7.13	**0.001**	0.062	0.921	0.36	0.11
After 8 weeks	46.14 ± 3.18^a^	44.37 ± 9.96^a^	44.70 ± 6.08^a^	43.92 ± 4.30^a^	40.05 ± 6.80	44.17 ± 14.68					

*Notes*: Data are presented as means ± standard deviation. Bold values are statistically significant.

Abbreviations: BF (%), body fat (%); BMI, body mass index; ES, effect size; FM, fat mass; *i*, intervention; LBM, lean body mass; LIS, leg isometric strength; SP, statistical power; *t*, time; WC, waist circumference; VO_2_max, maximal oxygen consumption; WHR, waist‐to‐hip ratio..

^a^ Significantly different from baseline (*p* < 0.05).

^b^ Significantly different from CON (*p* < 0.05).

**FIGURE 3:**
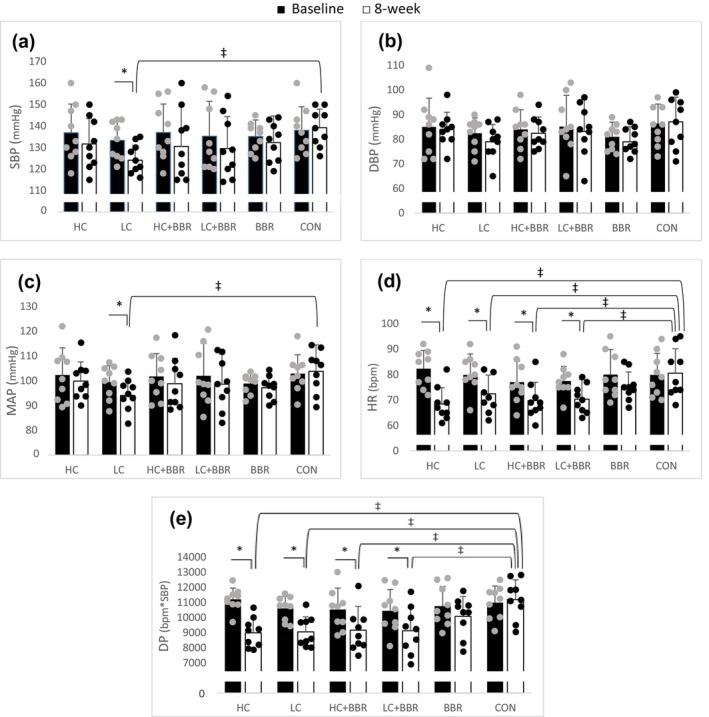
Changes in hemodynamic indices before and after 8 weeks of intervention across different groups. DBP, diastolic blood pressure; DP, Double product; HR, heart rate; MAP, mean atrial pressure; SBP, systolic blood pressure. * = significantly different compared to the baseline (*p* < 0.05). ‡ = significantly different from the CON (*p* < 0.05).

A significant improvement in VO2max was observed in all exercise groups when compared to CON, with no differences among the exercise groups themselves (all, *p* < 0.05). Additionally, significant improvements in leg isometric and hand grip strength were noted in all interventions compared to baseline (all, *p* < 0.05). However, there was no noticeable change was noted in lean body mass after these interventions. Heart rate and DP values declined after all interventions, except for BBR, when compared to CON (all, *p* < 0.05, 0.34 ≤ ES ≤0.37). The LC exercise program was the only one that resulted in a decrease in SBP (*p* = 0.02, ES = 0.12) and MAP (*p* = 0.02, ES = 12), with no change in DBP (Figure 3).

**FIGURE 4:**
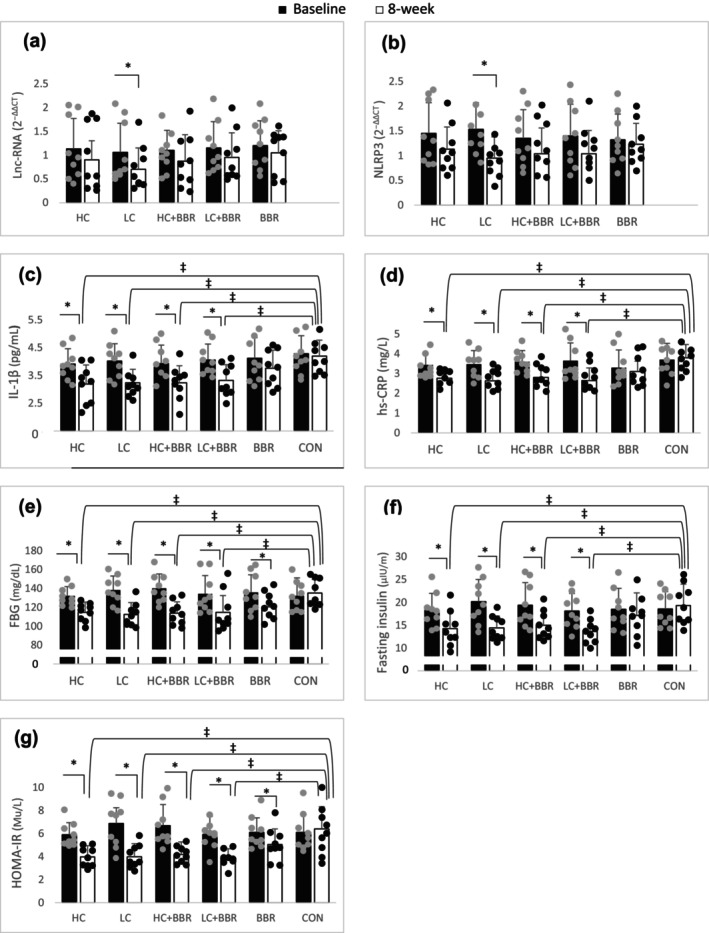
Changes of the gene expression and metabolic characteristics before and after 8 weeks of intervention across different groups. FBG, Fasting blood glucose; HOMA‐IR, homeostatic model assessment of insulin resistance; hs‐CRP, high sensitivity C‐reactive protein; Lnc‐RNA Imprinted Maternally Expressed Transcript (H19), long noncoding‐RNA H19; IL‐1β, Intelucin‐1β; NLRP3, NOD‐like receptor pyrin domain‐containing protein 3. * = significantly different from baseline (*p* < 0.05). ‡ = significantly different from CON (*p* < 0.05).

No significant change was found for the fold amount of NLRP3 and H19, although LC was the only exercise program that reduced these markers compared to baseline (both, *p* < 0.01, 0.14 ≤ ES ≤0.27; Figure 4). There was a significant drop in hs‐CRP, IL‐1β, insulin, fasting glucose, and HOMA‐IR after all exercise interventions compared to CON (all, *p* < 0.05, 0.34 ≤ ES ≤0.59; Figure 4), although the concentration of fasting blood glucose and HOMA‐IR decreased significantly in the BBR group compared to baseline (both, *p* < 0.01, 0.51 ≤ ES ≤0.54; Figure 4).

## 4 DISCUSSION

This is the first study to compare the effects of LC versus HC, with or without BBR, on selected inflammatory markers in middle‐aged men with overweight or obesity and prediabetes. In this study, all exercise interventions, with or without BBR consumption, were equally effective at improving fasting glucose, insulin, HOMA‐IR, IL‐1β, and hs‐CRP. However, LC was the only exercise protocol that led to positive changes in NLRP3 and H19. Fasting blood glucose and HOMA‐IR were the only markers that significantly reduced in the BBR group compared to baseline.

In the current study, LC was the only exercise program that resulted in a decrease in H19 levels. Explaining this result is challenging due to the limited availability of relevant studies. However, in a non‐exercise study, serum levels of H19 were found to be higher in diabetic and prediabetic patients compared to healthy individuals (Cheng et al., 2019; Fawzy et al., 2020). Thus, it can be suggested that the reduction of H19 following LC may indicate a positive trend toward improving the inflammatory status in prediabetic patients. It is noteworthy to mention that myocardial infarction led to a significant decrease in H19 levels in rats, which returned to normal after 4 weeks of moderate‐intensity aerobic training (Farsangi et al., 2021). This finding suggests that H19 may exhibit a beneficial role within a specific range; however, outside of that range, it may exert harmful effects. Another possible mechanism is that H19 may play a negative role in various tissues such as fat, skeletal muscle, and heart while potentially having a positive role in the blood, akin to interleukin 6. This is supported by the significant decrease in H19 observed in the skeletal muscle of diabetic patients, along with its implicated role in insulin signaling and glucose absorption deterioration (Gao et al., 2014).

Additionally, a significant decrease in NLRP3 was observed after LC only, while all exercise interventions resulted in reduced levels of hs‐CRP and IL‐1β. This finding contrasts with a previous study that demonstrated a significant decrease in NLRP3 levels following moderate aerobic training in young and healthy men, whereas these markers increased with higher intensity (Khakroo Abkenar et al., 2020). It was also noted that 12 weeks of low‐volume HIIT at 90% of VO2peak did not result in changes in hs‐CRP levels in patients with T2D (Way et al., 2020). One potential reason for these contradictions could stem from differences in training protocols, participant ages, and/or health statuses. In another study, rats were initially subjected to a high‐fat diet for 4 weeks, followed by 10 weeks of resistance and aerobic training (Mardare et al., 2016). The results indicated that NLRP3 and IL‐1β were significantly higher in adipose tissue in the high‐fat diet group compared to a group with a standard diet. However, aerobic training alone reversed IL‐1β to baseline levels, while both exercise programs led to a decline in NLRP3. Likewise, these markers exhibited a decrease following 8 weeks of endurance training at an intensity of 80% VO_2_peak, coupled with olive oil supplementation in obese male rats (Zare Damirchi et al., 2020). It can be inferred that factors associated with obesity may significantly influence the regulation of these variables, and the LC protocol (with longer recovery times) may have a greater capacity to reduce inflammatory biomarkers, particularly NLRP3. Importantly, the decrease in NLRP3 and H19 levels coincided with a reduction in SBP and MBP, suggesting a potential direct relationship between them.

In the current investigation, the administration of BBR alone or in conjunction with HIIT failed to yield significant enhancements in the selected inflammatory markers, despite its efficacy in reducing fasting blood glucose levels and HOMA‐IR. Notably, a recent meta‐analysis underscored the effectiveness of BBR in ameliorating insulin resistance markers, particularly fasting plasma glucose, among patients with T2D (Guo et al., 2021), mirroring the findings of our study. The observed improvement in insulin resistance markers may be attributed, in part, to the concurrent reduction in obesity indices, as evidenced by the ability of BBR consumption to decrease both body weight and body mass index within our study cohort. Notably, within hepatocytes, adipocytes, and myotubes, BBR has been observed to enhance glucose consumption and/or uptake independently of insulin stimulation (Ko et al., 2005; Yin et al., 2002; Yin, Gao, et al., 2008). This augmentation of glucose metabolism by BBR may arise from its ability to stimulate glycolysis, potentially in association with the inhibition of mitochondrial oxidation (Yin, Gao, et al., 2008). Additionally, berberine may exert its effects as an α‐glucosidase inhibitor, as indicated by its ability to inhibit disaccharidase activities and reduce glucose transportation across the intestinal epithelium (Pan et al., 2003).

Conversely, a meta‐analysis study revealed conflicting results regarding the impact of BBR on inflammatory markers. Specifically, while BBR supplementation was associated with a significant reduction in serum CRP levels among patients with metabolic syndrome, no significant effect was observed for IL‐1β levels (Lu et al., 2022). Further examination of Lu's study revealed two pertinent insights. Firstly, the reduction in CRP levels was predominantly observed in studies where BBR supplementation was administered in conjunction with metformin or other basic treatments, contrasting with the intervention protocol employed in our study. Secondly, participants in Lu's study exhibited higher baseline CRP levels, suggesting a potentially greater scope for improvement compared to our study cohort.

In this study, HR and DP decreased after all interventions, except for the BBR group, while the LC model demonstrated a significant improvement in SBP and MAP. This finding is supported by previous studies that have demonstrated significant reductions in SBP after 10 sessions of HIIT in individuals with prediabetes (Jung et al., 2015; Liu & Wang, 2021). Also, another study illustrated a significant decrease in SBP after 8 weeks of HIIT in men with overweight (Chin et al., 2020). On the contrary, SBP did not exhibit significant changes after 16 weeks of combined resistance and aerobic training in overweight individuals with T2D (Bonfante et al., 2022), contradicts the findings of this study. This discrepancy could be attributed to differences in training protocols or study populations. Therefore, it seems that the LC protocol alone has a positive effect on SBP in middle‐aged men with overweight or obesity and prediabetes.

This study represents the first exploration of the effects of the HC and LC exercise protocols, with or without the consumption of BBR, on CRP, IL‐1β, NLRP3, and H19 in middle‐aged men with overweight or obesity and prediabetes, which can be considered an advantage. However, further investigation into additional cytokines such as TNF‐α, NF‐kB, IL‐6, and IFN‐γ could provide deeper insights into the effects of non‐pharmacological interventions on inflammation. A limitation of this study is that the training programs were conducted online due to complications arising from COVID‐19, which hindered accurate control over these programs. Additionally, we recommend a larger sample size to effectively detect significant intervention effects on selected biomarkers.

## 5 CONCLUSION

In summary, the results of this study revealed that both LC and HC protocols had similar positive effects on most selected parameters in middle‐aged men with overweight or obesity and prediabetes, and consuming BBR did not provide additional benefits. These findings also indicated that an 8‐week exercise period may not be sufficient to detect potential differences between these interventions. However, the LC model was the only exercise program that exhibited more beneficial effects in improving H19, NLRP3, and blood pressure. Combining it with BBR was found to be more effective in reducing obesity indices compared to the other model. Therefore, it can be concluded that the consumption of BBR with LC exercise represents a viable strategy, at least for improving certain body composition parameters. Finally, given the scarcity of exercise studies examining these gene expression markers, particularly in different tissues, we recommend further investigation into these markers following longer exercise programs, with or without consuming BBR, in future studies.

## FUNDING INFORMATION

None.

## CONFLICT OF INTEREST STATEMENT

The authors declare that no conflict of interest would prejudice their impartiality.

## ETHICS STATEMENT

This study was approved by the Research Ethics Committee of Mazandaran University (IR.UMZ.REC.1401.011) in accordance with the Helsinki Declaration.

## Data Availability

Data generated or analyzed during the present study that support the findings are available from the corresponding author upon reasonable request.
